# Local Epidemics Gone Viral: Evolution and Diffusion of the Italian HIV-1 Recombinant Form CRF60_BC

**DOI:** 10.3389/fmicb.2019.00769

**Published:** 2019-04-12

**Authors:** Alessia Lai, Francesco Roberto Simonetti, Gaetano Brindicci, Annalisa Bergna, Simona Di Giambenedetto, Gaetana Sterrantino, Cristina Mussini, Stefano Menzo, Patrizia Bagnarelli, Maurizio Zazzi, Gioacchino Angarano, Massimo Galli, Laura Monno, Claudia Balotta

**Affiliations:** ^1^Department of Biomedical and Clinical Sciences L. Sacco, University of Milan, Milan, Italy; ^2^Department of Medicine, Johns Hopkins University School of Medicine, Baltimore, MD, United States; ^3^Clinic of Infectious Diseases, University of Bari Aldo Moro, Bari, Italy; ^4^Institute of Infectious Diseases, Catholic University of Sacred Heart, Rome, Italy; ^5^Division of Tropical and Infectious Diseases, Careggi Hospital, Florence, Italy; ^6^Clinic of Infectious Diseases, University of Modena and Reggio Emilia, Modena, Italy; ^7^Unit of Virology, Azienda Ospedaliero-Universitaria ‘Ospedali Riuniti’, Torrette, Italy; ^8^Department of Medical Biotechnologies, University of Siena, Siena, Italy

**Keywords:** HIV-1 recombinant forms, HIV-1 molecular epidemiology, HIV-1 evolution, HIV-1 outbreak, second generation recombinants

## Abstract

The molecular epidemiology of HIV-1 in Italy is becoming increasingly complex, mainly due to the spread of non-B subtypes and the emergence of new recombinant forms. We previously characterized the outbreak of the first Italian circulating recombinant form (CRF60_BC), occurring among young MSM living in Apulia between the years 2009 and 2011. Here we show a 5-year follow-up surveillance to trace the evolution of CRF60_BC and to investigate its further spread in Italy. We collected additional sequences and clinical data from patients harboring CRF60_BC, enrolled at the Infectious Diseases Clinic of the University of Bari. In addition to the 24 previously identified sequences, we retrieved 27 CRF60_BC sequences from patients residing in Apulia, whose epidemiological and clinical features did not differ from those of the initial outbreak, i.e., the Italian origin, young age at HIV diagnosis (median: 24 years; range: 18–37), MSM risk factor (23/25, 92%) and recent infection (from 2008 to 2017). Sequence analysis revealed a growing overall nucleotide diversity, with few nucleotide changes that were fixed over time. Twenty-seven additional sequences were detected across Italy, spanning multiple distant regions. Using a BLAST search, we also identified a CRF60_BC sequence isolated in United Kingdom in 2013. Three patients harbored a unique second generation recombinant form in which CRF60_BC was one of the parental strains. Our data show that CRF60_BC gained epidemic importance, spreading among young MSM in multiple Italian regions and increasing its population size in few years, as the number of sequences identified so far has triplicated since our first report. The observed further divergence of CRF60_BC is likely due to evolutionary bottlenecks and host adaptation during transmission chains. Of note, we detected three second-generation recombinants, further supporting a widespread circulation of CRF60_BC and the increasing complexity of the HIV-1 epidemic in Italy.

## Introduction

The human immunodeficiency virus (HIV-1) is a well-known moving target. HIV-1 high variability and rapid adaptation impose an additional challenge to its diagnosis, treatment, and prevention (Hemelaar, 2013). However, HIV-1 evolutionary potential can be harnessed to understand its complex dynamics, both at cellular and population level. Thanks to viral diversity, molecular epidemiology allows to track HIV-1 global and local epidemics and provide insights on how to control their further diffusion (Hemelaar, 2012).

The pandemic group M spread worldwide and differentiated over time into nine distinct subtypes (A–D, F–H, J, and K) and 8 sub-subtypes (A1–A6, F1, and F2) (Peeters et al., 2014). In addition to HIV-1 error prone reverse transcriptase and high replicative potential, recombination plays a key role in its variability (Hu and Hughes, 2012). Due to recombination, when two or more subtypes co-circulate among high risk groups in the same geographical area, unique recombinant forms (URFs) emerge and, if they are detected in at least three epidemiologically unrelated patients, they gain the role of circulating recombinant forms (CRFs) (Robertson et al., 1995). The number of identified CRFs has grown dramatically; currently, 98 CRFs have been characterized and their virological and epidemiological data are available at the Los Alamos HIV database^1^. The first CRFs have been found mainly in central Africa and South America, where multiple subtypes co-circulate at high prevalence (Thomson et al., 2002). In the past few years, some of these recombinant progenies have caused outbreaks (Monno et al., 2012; Gonzalez-Domenech et al., 2018) and in some cases have shown an increased fitness (Rawson et al., 2018). For example, the Cuban CRF19_cpx, has been associated with a higher viral load, a more rapid progression to AIDS (Kouri et al., 2015) and antiretroviral resistance mutations (Kouri et al., 2014). HIV-1 M chimeric forms, URFs and CRFs altogether, are estimated to represent at least 20% of infections globally, with CRF01_AE and CRF02_AG being the most prevalent, accounting for about 13% of all infections (Osmanov et al., 2002). Despite their origin, some CRFs gained epidemiological relevance in previously B subtype-restricted countries with lower HIV-1 prevalence. Within the last years, multiple new CRFs have been identified in European countries such as Russia (Liitsola et al., 1998; Lukashov et al., 1999; Baryshev et al., 2014), Portugal, Spain (Delgado et al., 2002; Fernandez-Garcia et al., 2016), Italy (Simonetti et al., 2014), United Kingdom (Foster et al., 2014), Luxembourg (Struck et al., 2015), and France (Leoz et al., 2013; Recordon-Pinson et al., 2018).

The molecular epidemiology of HIV-1 in Italy, previously a B-restricted area, has become complex, as migratory waves and travels favored the penetration of HIV-1 forms from countries where non-B subtypes prevail (Lai et al., 2012, 2014a,b, 2016). A comprehensive study reported that non-B variants increased dramatically since the beginning of the epidemic with a prevalence of 15.8 and 29.7% of all HIV-1 diagnoses from 2006 to 2016 (Rossetti et al., 2018). After subtype B, the three most abundant pure subtypes in Italy were F, A and C, and CRFs represented almost 50% of non-B forms (Lai et al., 2010). Monno et al. (2012) reported a sudden HIV-1 outbreak among young men-who-have-sex-with-men (MSM) residing in Apulia, a region of southern Italy. This outbreak was caused by an inter-subtype recombinant, which was identified as the first Italian CRF, named CRF60_BC, by a subsequent study (Simonetti et al., 2014). This recombinant form is mostly composed of subtype C with the exception of three fragments from a parental B subtype which covered a part of the 5′ LTR (HXB2 positions 0–462), a small region of RT *pol* gene (HXB2 positions 2,804–3,037) and the last part of *env* gene. *Nef* gene and most of the 3′ LTR (HXB2 positions 8,662–9,548) were also subtype B. At the time of its characterization, the outbreak involved 22 individuals residing in Apulia and additional few patients from Emilia-Romagna, Tuscany and Sicily, all diagnosed between 2009 and 2011.

Here, we present a follow up study in which we investigated the further circulation and evolution of CRF60_BC. We combined epidemiological data with phylogenetic analyses on sequences retrieved in Apulia and those available from a nation-wide database. We observed an increasing population size and evolution of CRF60_BC, whose spread resulted also in novel recombination events. Overall, the present study portrays an increasingly complex and dynamic landscape of the HIV-1 Italian epidemic.

## Materials and Methods

### Patients

We collected sequences and clinical data from patients, harboring CRF60_BC, enrolled at the Infectious Diseases Clinic of the University of Bari. We found additional sequences from the national ARCA cohort. Putative CRF60_BC sequences were searched among those assigned to subtype C or CRF31_BC in *pol* gene, according to a preliminary subtyping based on BLAST. As the CRF60_BC contains a small portion of B subtype in this region, the BLAST search lead to a subtype misclassification. Sequences were retrieved from the genotypic drug resistance assay performed by the local Services at diagnosis or prior to the start of therapy or at treatment failure. Only the first available resistance genotype was considered for downstream analyses. Genotypic resistance from plasma HIV-RNA was determined by bulk Sanger sequencing using commercially available or homebrew methods, depending on the contributing laboratory.

ARCA is an observational HIV cohort approved by the Regional Ethical Committee of Tuscany (Comitato Etico Area Vasta Toscana Sudest). The study was conducted in accordance with the 1964 Declaration of Helsinki and the ethical standards of the Italian Ministry of Health. The national ARCA database, currently contained demographic and viroimmunological data of 40,654 patients enrolled at 90 Clinical Centers, and at list one sequence before the start of antiretroviral therapy for all subjects. All patients belonging to the ARCA database provided informed consent to have their anonymized data stored on a central server and used for research studies. For all patients, epidemiological data (gender, risk category, country of origin, date of diagnosis, and age) were collected by physicians from medical records and then included in the databases together with virological, immunological, and treatment information through standard procedures cleared by the Southeast Tuscany Ethics Committee. When available, analyses were conducted on multiple HIV-1 regions: protease (PRO), reverse transcriptase (RT), integrase (INT), gp120 and gp41. The estimated time from infection was calculated by computing the fraction of ambiguous nucleotides in the PRO-RT sequences for all patients, as previously reported (Kouyos et al., 2011).

### Alignment and Phylogenetic Analysis

BioEdit version 7.2.6.1^2^ was used to manually align query sequences with pure subtypes and CRF reference sequences. The alignment of a comprehensive list of reference sequences of different subtypes (A1, A2, B, C, D, F1, F2, G, H, J, K) was retrieved from the Los Alamos HIV database ^3^. In particular, we included 47, 37, 36, and 37 reference sequences for PRO/RT, INT, gp120, and gp41 fragments, respectively. The evolutionary model that best fitted the data was selected using the information criterion implemented in JmodelTest v2.1.7 (Posada, 2008). We then conducted phylogenetic analyses for subtyping on more than 10,000 sequences from the national ARCA cohort with maximum likelihood approach implemented in MEGA v7.0 program to retrieve additional CRF60_BC circulating in Italy.

The monophyletic nature of the sequences was evaluated by means of the MrBayes program (Huelsenbeck and Ronquist, 2001) using a general-time reversible (GTR) or Hasegawa-Kishino-Yano (HKY) model of nucleotide substitution, a proportion of invariant sites, and gamma distributed rates among sites for PRO, RT, gp120 and gp41 regions, respectively. For INT region HKY plus a proportion of invariant sites was used. The final alignment encompassed 1,259, 1,029, 514, and 858 nucleotides for PRO/RT, gp120, gp41, and INT regions, respectively.

A Markov Chain Monte Carlo (MCMC) search was made for 2 × 10^6^ generations using tree sampling every 100^th^ generation and a highly conservative burn-in fraction of 50% to obtain a better signal of convergence. The analysis was run until reaching the average standard deviation < 0.01. Statistical support for specific clades was obtained by calculating the posterior probability of each monophyletic clade, and a posterior consensus tree was generated after a 50% burn-in.

Pairwise distances were measured with MEGA v7.0 (Kumar et al., 2016), as the p-distance of each taxa from the CRF60_BC sequence harbored by the patient with the oldest time of diagnosis (patient BAV275, year 2005), with variance estimation performed using 1,000 bootstrap replicates. Spearman test of correlation between genetic distance and time of diagnosis was conducted with GraphPad Prism v7.0. Nucleotide differences across CFR60_BC sequences from Apulia were plotted with Highlighter (Keele et al., 2008)^4^, with sequences sorted according to tree topology; ambiguous IUPAC codes were excluded from the graph.

Similarity and bootscanning plots implemented in Simplot software v3.5.2^5^ were used to identify the subtypes involved in the recombination and their breakpoints to reconstruct recombination pattern. Maximum Likelihood phylogenetic trees were constructed for individual segments identified within each breakpoint with bootstrapping on 1,000 replicates using MEGA v7.0 program. Recombination was also characterized by split decomposition method and neighbor-net network generated with SplitsTree program^6^. Final trees were visualized and annotated with FigTree v1.4.0^7^.

HIV BLAST tool^8^ was used to retrieve sequences showing high similarity and the same BC mosaic pattern within the Los Alamos HIV Sequence Database. The prediction of HIV-1 co-receptor usage was performed using Geno2Pheno^9^.

### Phylodynamic Analysis

Dated trees of the CRF60_BC portions (INT, gp120, and gp41) not containing recombination breakpoints were estimated by BEAST software v1.8.4 (Drummond and Rambaut, 2007) using the previously selected models. For this purpose, only sequences with known date of collection were used. Chains were run for 10–30 million generations with sampling every 1,000–3,000 generations. The results were visualized in Tracer v1.5 and uncertainty in the estimates was indicated by 95% highest probability density (HPD) intervals. Convergence was assessed based on the ESS (effective-sample size) value and only parameter estimates with ESS > 300 were accepted. The maximum clade credibility (MCC) tree was then selected from the posterior tree distribution using TreeAnnotator v1.8.4 available in the BEAST software package. Final trees were visualized and edited with FigTree v1.4.0.

## Results

### Evolution of the CRF60_BC Outbreak in Apulia

**FIGURE 1:**
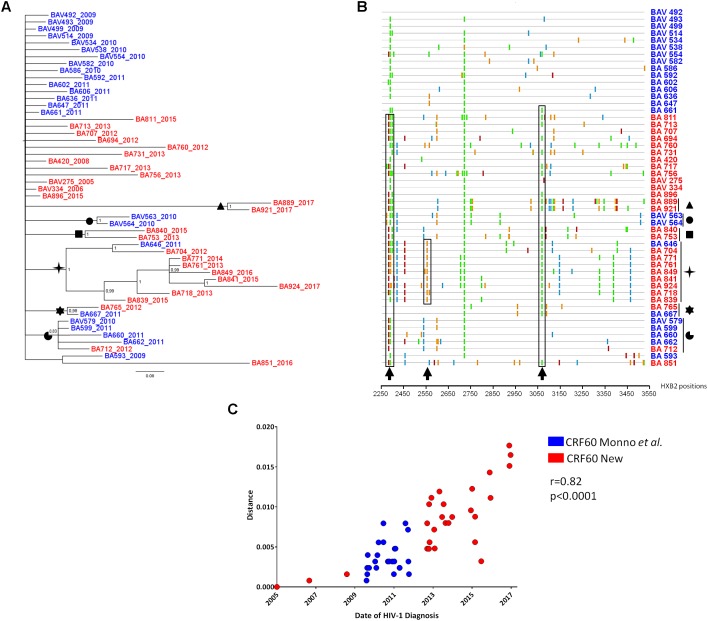
Evolution of CRF60_BC PRO-RT sequences from Apulia. **(A)** Bayesian tree of PRO-RT Apulian sequences obtained by MrBayes program. Names of sequences include the year of sampling. The newly identified sequences are shown in red, while those previously described are shown in blue. Posterior probabilities > 0.8 are shown at nodes. **(B)** Highlighter plot of nucleotide mismatches in the protease and RT regions; sequences are sorted based on the topology of the tree in **(A)**, with the top sequence used as the master. Arrows and boxes indicate non-synonymous changes occurring in known epitopes under cytotoxic T lymphocyte (CTL) pressure. Symbols indicated the sequence clusters present in the tree. **(C)** Correlation plot of genetic pairwise distances of HIV-1 diagnosis over time.

We first investigated whether the CRF60_BC had spread further locally, where the original outbreak occurred in 2009–2011. In addition to the 24 sequences previously described (Monno et al., 2012), we retrieved from local clinical centers, 27 new CRF60_BC sequences from patients residing in Apulia. The epidemiological and clinical features of the patients carrying this variant did not differ from those of the initial outbreak (Table 1). Similarly to the patients previously described, these individuals were Italian (100%) with young age at HIV-1 diagnosis (median: 26 years; range: 18–53) and all but one were MSM (25/26, 96%). The majority of these patients had a recent diagnosis in 2012–2017 and a CD4+ T-cell count at diagnosis above 350 cells/μl in 23 out 27 cases (median: 506 cells/μl; range: 46–1,187). Three subjects (BAV718, BAV731, BAV851) had levels of plasma HIV-RNA above 10^6^ copies/ml, suggesting that they were diagnosed before reaching a viral set point. These data support that most of the patients carrying the CRF60_BC variant were infected recently, during the last few years after the initial outbreak. However, we identified three subjects, BAV275, BAV334, BAV420, whose HIV-1 infection occurred before the initial outbreak, as their diagnosis dated back to 2005, 2006, and 2008, respectively (Simonetti et al., 2014). These patients were recorded in the ARCA database after the characterization of CRF60_BC. Last negative test was available for 10 subjects and confirmed a seroconversion time within 12 months in six cases. The analysis of the fraction of ambiguous nucleotides provided strong evidence of a recent infection (<1 year) for 77.8% (*n* = 21) of subjects. The Bayesian tree of patients from Apulia is shown in Figure 1A. Twelve out 27 (44.4%) recently identified strains showed high similarity with the majority of previous described sequences (15/24, 62.5%). We identified six significant clusters supported by a posterior probability higher than 0.80. Two of them corresponded to known partners or epidemiological links (pp = 1, BA889-BA921, BAV563-BAV564). The largest cluster (pp = 1) involved one of the sequences identified in 2011 and eight newly detected strains (year of diagnosis ranging between 2012 and 2017). Another large cluster (pp = 0.83) included four previously described CRF60_BC sequences and one sequence identified in 2012. The remaining two clusters (pp = 1 and pp = 0.98, respectively) involved two strains each; the first included two new sequences (BA840 and BA753) and the second a previously described strain (BA667) with a new one (BA765).

**Table 1:** Characteristics of patients harboring CRF60_BC.

Characteristics	CRF60_BC (Monno et al., 2012) (*n* = 24)	CRF60_BC new from Apulia (*n* = 27)	CRF60_BC new outside Apulia (*n* = 27)
**Region [%; (n)]**			
Apulia	100 (24)	100 (27)	–
Emilia	–	–	22.2 (6)
Tuscany	–	–	22.2 (6)
Sicilia	–	–	3.7 (1)
Marche	–	–	3.7 (1)
Liguria	–	–	3.7 (1)
Lombardy	–	–	3.7 (1)
Lazio	–	–	29.6 (8)
Trentino	–	–	3.7 (1)
Piemonte	–	–	3.7 (1)
Umbria	–	–	3.7 (1)
Unknown	–	–	3.7 (1)
**Gender [%; (n)]**			
Male	91.7 (22)	100 (27)	81.4 (22)
Unknown	8.3 (2)	–	14.8 (4)
**Age at diagnosis [years]**			
Median [IQR] (n)	25 [25–25](24)	26 [22.5–29](27)	25 [25–34.5](12)
**Year of diagnosis**			
Median [IQR] (n)	2010 [2010–2011](24)	2013 [2012–2015](27)	2013.5 [2013–2014](11)
**Country of origin [%; (n)]**			
Italy	100 (24)	100 (27)	59.2 (16)
Cote d’Avoire	–	–	3.7 (1)
United Kingdom	–	–	3.7 (1)
Unknown	–	–	33.3 (9)
**Modality of transmission**			
Men-who-have-sex-with-men	83.3 (20)	92.6 (25)	40.7 (11)
Other	16.7 (4)	3.7 (1)	3.7 (1)
Unknown	–	3.7 (1)	55.5 (15)
**CD4 count^∗^[cell/μl]**			
Median [IQR] (n)	465.5 [393.3–659.5](24)	506 [420–672.5](27)	570 [467–637](13)
**pVL at sequencing [copies/mL]**			
Median [IQR] (n)	7,700 [3,150–32,500](24)	8,000 [4,700–26,000](27)	24,197 [7,528–56,582.5](12)
**Time from seroconversion [%; (n)]**			
Seroconversion within 12 month	54.5 (6)	60 (6)	66.7 (2)
Chronic	45.5 (5)	40 (4)	33.3 (1)
**Estimated age of infection^∗∗^[%; (n)]**			
<1 year	75 (18)	77.8 (21)	40.7 (11)
>1 year	25 (6)	22.2 (6)	59.3 (16)

*^∗^At the time of sequencing; ^∗∗^As in Kouyos et al. (2011).*

The emergence of new cases of HIV-1 infection due to CRF60_BC could be explained both by an increase of diagnoses after the initial outbreak (more effective sampling) and actual onward dissemination. To further understand whether CRF60_BC has been involved in ongoing transmission chains, we looked at sequence diversity and accumulation of mutations over time (Figure 1B,C). We observed the emergence of non-synonymous mutations strongly associated with CD8+ T-cell immune pressure against epitopes in *gag* (HXB2 position 498) and *pol* (HXB2 positions 69, 72, 128, and 314) (Addo et al., 2003; Allen et al., 2005). Some mutations were fixed over time, like in the case of the large transmission cluster encompassing sequences from 2011 to 2017, in which we observed reversion to subtype B consensus of a known conserved *pol* epitope (HXB2 *pol* 314) associated with HLA-I responses (Addo et al., 2003; Apps et al., 2013). In addition, when we estimated pairwise distances of all sequences (*n* = 47) from the oldest isolate (2005), we detected a growing overall diversity that strongly correlated with time of HIV-1 diagnosis (*r* = 0.82, *p* < 0.0001), further supporting ongoing evolution of CRF60_BC over time.

### Diffusion of CRF60_BC Outside Apulia

**FIGURE 2:**
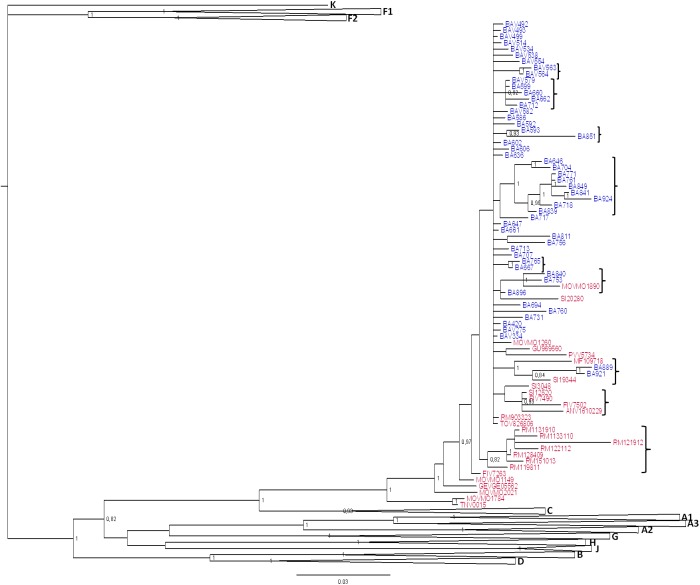
Phylogenetic tree of CRF60_BC PRO-RT sequences detected in and outside of Apulia. Bayesian tree of PRO-RT CRF60_BC obtained using MrBayes program. Posterior probabilities > 0.8 are shown at nodes. Sequences in blue are from Apulia region, in red those retrieved from other Italian regions and two sequences detected using BLAST search. The brackets highlight the significant sub-clades.

To investigate the further diffusion of CRF60_BC, we conducted subtyping analyses on the ARCA database retrieving 25 sequences belonging to CRF60_BC (data not shown). Figure 2 shows the Bayesian tree containing 78 sequences: the original and the newly identified sequences from Apulia (total, *n* = 51), sequences from different Italian regions obtained from the ARCA database (*n* = 25) and two sequences detected using BLAST search (GU969560 and MF109718).

Sequences assigned to CRF60_BC formed a highly supported cluster (pp = 1). Twenty-five sequences were detected across the Italian territory (Rome, Florence, Modena, Arezzo, Fermo, Pavia, Genoa, Perugia, Trento, and Turin). Some sub clusters were observed, containing 2 to 9 strains. Sequences from Apulia segregated alone, except for four sequences. Two strains (BA840 and BA753) grouped significantly (pp = 1) with a sequence from Modena (MOV-MO-1890) while the remaining two (BA889 and BA921) formed a sub cluster with a strain from Perugia (SI-19344) and a sequence detected from a patient in United Kingdom (MF109718). This latter sequence, retrieved by BLAST, had no demographic information apart from the sampling date (November 2013), preventing us to rule out whether the patient acquired the infection in his/her country of origin or abroad. All sequences from Rome (*n* = 7), with the exception of RM9-03323, formed a significant (pp = 0.82) sub cluster. Three sequences from Tuscany (one from Siena: SI-12520 and two from Florence: FIV-7490 and FIV-7502) significantly grouped (pp = 0.95) with a sequence from central Italy (ANV-1610-229). A second strain was found by BLAST, isolated in Sicily from an African man with unknown risk factors.

The epidemiological characteristics of subjects carrying CRF60_BC residing outside Apulia (Table 1) did not differ substantially from those residing in Apulia. All but one patients were males (22/23, 96%), with a median age of 25 years (range: 21–52), diagnosed from 2009 to 2014 and, for subjects with known risk factors, predominantly MSM (13/14, 92.8%). The last negative HIV-1 test, available only for three patients, suggested a recent seroconversion (less than 12 months) in two cases. However, the analysis of the fraction of ambiguous nucleotides indicated that the majority of patients (59.3%, *n* = 16) had been infected for more than 1 year.

### Emergence of Second Generation Recombinant Forms

**FIGURE 3:**
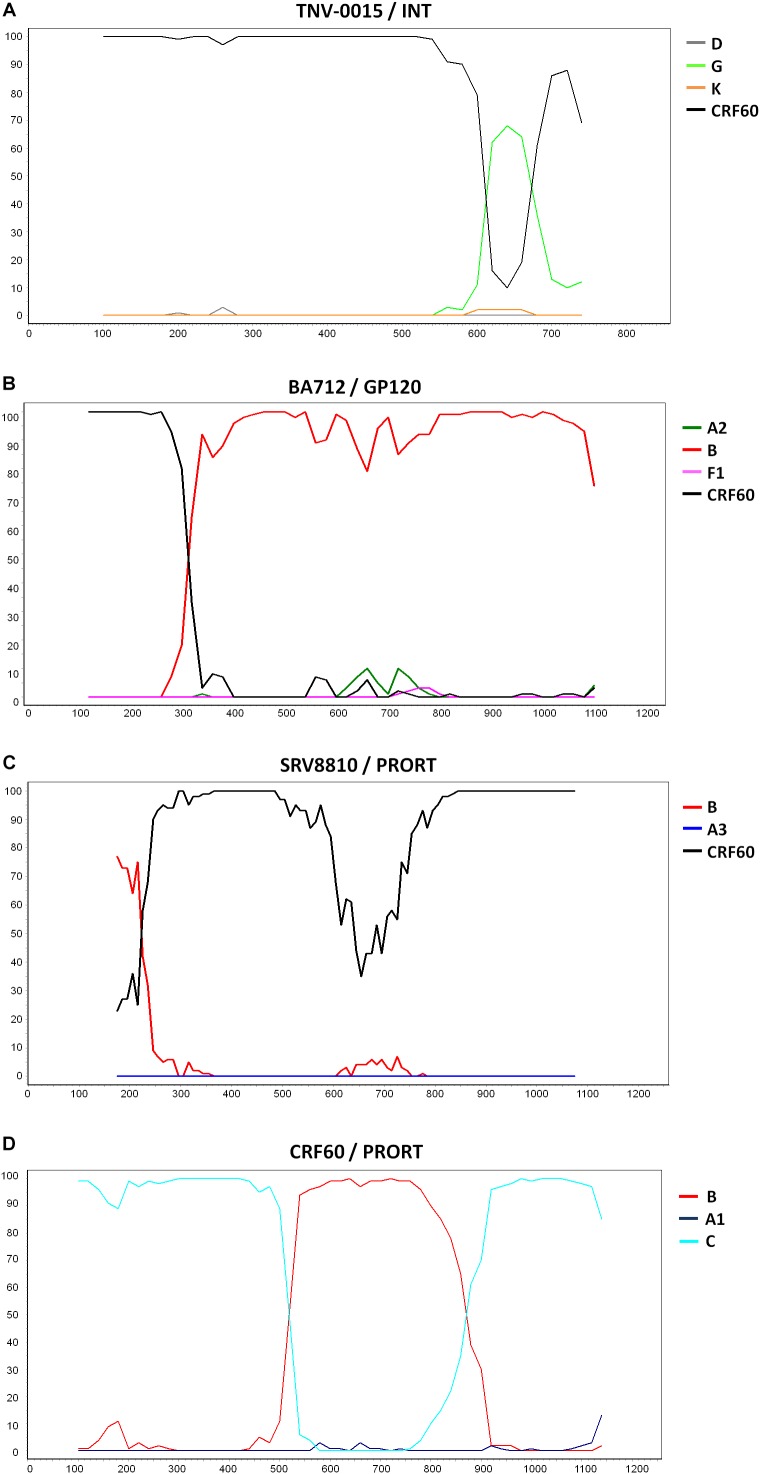
Recombination sites of 2^nd^ generation recombinants involving CRF60_BC. Bootscan analyses of the three unique recombinant forms (URF) in which CRF60_BC was one of the parental strains. Bootscanning plot is performed with the Simplot program using a window between 200 and 350 bp and step size between 10 and 20 bp. **(A)** Bootscan of TNV-0015 in the integrase portion. **(B)** Bootscan of BA712 in the GP120 portion. **(C)** Bootscan of SRV8810 in the PRO-RT portion. **(D)** Bootscan of CRF60_BC in the PRO-RT portion.

Three patients harbored URFs in which CRF60_BC was one of the parental strains, as indicated by the phylogenetic trees shown in Supplementary Figures S1, S2, S3A. Figure 3 shows the bootscan analysis of these sequences and the CRF60_BC bootscan of *pol* region. Protease and RT regions of two of these sequences clustered significantly with CRF60_BC confirming the recombinant BC pattern, but different recombination patterns were detected in INT for strain TNV-0015 (Figure 3A) and in gp120 for BA712, in which CRF60_BC is subtype C. The bootscan analysis identified a CRF60/G mosaic pattern with two breakpoints at positions 4,848 and 4,907 (as mapped to HXB2 reference) with a 60 base-pair fragment of subtype G in the middle of the integrase region. Recombination analysis on BA712 identified a CRF60/B recombinant with one breakpoint at position 6,710 (Figure 3B). This recombination structure in gp120 resulted in a switch in co-receptor viral tropism from CCR5 to CXCR4. The sequence SRV8810, showed a B/CFR60 mosaicism in the PRO-RT region (Figure 3C) with a portion belonging to B subtype at the 3′ end of protease (position 2,489).

Supplementary Figures S1, S2, S3B,C show the maximum likelihood trees for individual segments identified within each breakpoint confirming the recombination pattern for all recombinants. Despite the presence of TNV-0015 (Supplementary Figure S1C) and SRV8810 (Supplementary Figure S3B) in subtype G and B clades respectively, these clusters are not bootstrap supported due to the limited length of the analyzed fragments.

### Phylodynamic Analysis

Evolutionary population dynamics were estimated based on the data sets of non-recombinant regions (gp120 and gp41) of CRF60_BC sequences from Apulia with known data. The INT portion was not evaluated due to the limited availability of sequences. After testing different coalescent priors (constant population size, exponential growth, logistic growth, and Bayesian skyline plot), using both strict and relaxed molecular clock models, the Bayes factor (BF) analysis showed that the relaxed lognormal molecular clock fitted the data better than the strict clock model (2lnBF = 114.5) and the Bayesian skyline plot fitted the data better than the other models. The estimated mean rate of evolution was 7 × 10^−3^ substitutions per site per year (s/s/y) (95%HPD: 5.1 × 10^−3^–9.2 × 10^−3^) and 6.2 × 10^−3^ s/s/y (95%HPD: 3.4 × 10^−3^–9.3 × 10^−3^) for the gp120 and gp41, respectively. The time of the most recent common ancestor (tMRCA) corresponding to the root of the trees was estimated to be 11.4 years before the latest sampling data for both regions (95%HPD: 11–12.85 years), corresponding to mid 2004, very close to the year of diagnosis of the oldest sequence in our data set (BAV275, 2005).

The Bayesian Skyline plot (Supplementary Figure S4) shows the changes in population size at different times from the root of the tree to the time of the most recent samples (year 2016 and 2017). The estimated effective number of infections exponentially grew from the middle of 2006 to 2011, when the initial outbreak was identified; after this period, the curve reached a plateau and remained relatively constant until the latest calendar years.

## Discussion

Although the first CRFs have been found in Africa and mainly Central Africa, inter-subtype recombination events have also occurred in other continents, leading to the spread of new CRFs. Several CRFs have been described in Europe, as different non-B subtypes circulate together with the founder B clade and are detected at increasing prevalence almost in all countries (Tebit and Arts, 2011). Several European CRFs show a mosaic structure due to multiple recombination events with different parental subtypes; however, the B clade is the most frequent parental subtype. Indeed, HIV-1 superinfection, which underlies the recombination events, has been frequently reported in MSM due to the numerous risk contacts (Redd et al., 2013). As the B variant had a founder effect within intravenous drug users in some European countries including Italy (Lukashov et al., 1996), some of these CRFs (CRF03_AB and CRF14_BG) mostly segregate in this specific high risk population (Hemelaar, 2013). Some recombinant forms (i.e., CRF05_DF and CRF47_BF) have generated limited epidemics both in frequency and regional dispersion (Fernandez-Garcia et al., 2010), while others (CRF03_AB and CRF14_BG) have had a relevant impact on the HIV-1 epidemic, both in the countries of first detection and neighboring countries (Liitsola et al., 1998; Delgado et al., 2002).

We previously described a new CRF epidemic in a single Italian region (Apulia) mainly involving young MSM seeking sexual partners through on-line dating websites, who acquired HIV-1 infection in 2009–2011 and were diagnosed soon after seroconversion (Monno et al., 2012; Simonetti et al., 2014). The subtype C regions included in CRF60_BC are highly similar to the South American variant, suggesting that CRF60_BC originated in South America where subtypes B and C largely co-circulate and other BC inter-subtype recombinants have been documented (Hemelaar, 2013). Alternatively, CRF60_BC might have originated in Italy where, subtype C, mostly harbored by recent immigrants and Italian individuals who traveled to endemic regions, accounts for 13% of non-B variants (Lai et al., 2010). Additionally, our group recently conducted a phylogeographic analysis revealing that the occurrence of subtype C in Italy is related to the South American variant, likely due to transmission chains among MSM (Lai et al., 2014a). To follow-up the epidemic potential of the originally described CRF60_BC, we traced its spread through a large Italian database collecting HIV-1 sequences prospectively.

CRF60_BC was originally limited to the Apulia region but then reached distant regions of Italy. Increased sequence diversity after the initial outbreak suggests evolutionary bottlenecks along the transmission chains and adaptation to the host immune pressure. This observation is supported by the fact that some mutations, predominantly non-synonymous mutations, were fixed overtime and that the growing diversity among strains significantly correlates with the time of HIV-1 diagnosis. In this study, most CRF60_BC variants circulate among young MSM who recently acquired HIV-1 infection during the 2005–2017 period. These characteristics did not differ from those reported for patients described in the initial outbreak, indicating that CRF60_BC circulation is actually closely linked to MSM. Due to the higher prevalence in this population and HIV-1 increasing genetic diversity, the MSM population might become a new recombination hotspot, as observed in previous studies in China reporting several CRFs among MSM population (Han et al., 2013; Wu et al., 2013; Zhang et al., 2014). Noteworthy, the number of sequences identified so far is three fold higher compared to our first report.

In addition, we detected three second-generation recombinants which further support a widespread circulation of CRF60_BC, all of which showed recombination in different portions of the HIV-1 genome. In one case, the recombination pattern involved subtype G, an uncommon subtype that is present only at 6% of non-B subtype strains (Lai et al., 2010). In another case, recombination in gp120 entailed the coreceptor switch to CXCR4, despite the recent acquisition of HIV-1 infection in 2012 confirmed by CD4+ T-cell count and viral load measure at diagnosis. By identifying new recombinant forms with a BC pattern in MSM, we demonstrated that these individuals are likely to represent a preferential group that may give rise to HIV-1 recombinants.

The finding of a patient carrying CRF60_BC diagnosed in 2005 indicates that the circulation of this strain probably began in the early 2000s. The phylodinamic analysis set the origin of CRF60_BC in the mid 2004 (range: 2003–2005) suggesting that this subject could be one of the first cases of CRF60_BC. The exponential growth of infections sustained by CRF60_BC was observed in 2006–2011 period when most patients were identified, suggesting a rapid spread among young MSM with recent infection through Italy.

This study has some limitations. First, information related to studied patients was not always available or complete. Second, the data set in the current study may not be representative of the entire country and we may have missed a number of recently infected individuals present in additional local networks. Regarding the unique recombinants, additional studies involving single genome amplification and full-length genome sequencing, are required to extend the characterization of the recombination patterns and to exclude the role of superinfection. Despite our analysis might represent an underestimate, our data strongly suggest that CRF60_BC gained epidemic importance, spreading in multiple Italian regions and increasing its population size in very few years (2007–2013), among young MSM.

This is valuable information for public health agencies developing strategies to prevent the HIV-1 epidemic to spread into a more complex epidemiological landscape. In addition, our results further highlight the need for better prevention campaigns in young MSM, who represent a population with a poor control of HIV-1 transmission (Volz et al., 2018). Finally, future studies should continue to focus on transmission clusters and pay special attention to recombinant forms, as these variants not only reflect the changing landscape of HIV-1 diversity, but can also unveil the onset of new epidemic bursts.

## Ethics Statement

ARCA is an observational HIV cohort approved by the Regional Ethical Committee of Tuscany (Comitato Etico Area Vasta Toscana Sudest). The study was conducted in accordance with the 1964 Declaration of Helsinki and the ethical standards of the Italian Ministry of Health. Patients included on ARCA database signed an informed consent and agreed to have their anonymized data stored on a central server and used for research studies.

## Author Contributions

AL and FS conceived and designed the study. GB, GA, and LM collected and analyzed the epidemiological and viral data of patients from Apulia. SG, GS, CM, MZ, SM, and PB provided epidemiological and viral data of patients from ARCA database. AL, FS, AB, and CB wrote the first draft of the manuscript. All authors contributed to manuscript revision, read and approved the submitted version.

## Conflict of Interest Statement

The authors declare that the research was conducted in the absence of any commercial or financial relationships that could be construed as a potential conflict of interest. The reviewer MC declared past co-authorships with two of the authors AL and MG to the handling Editor.

## Footnotes

1 https://www.hiv.lanl.gov/content/sequence/HIV/CRFs/CRFs.html

2 http://www.mbio.ncsu.edu/bioedit/bioedit.html

3 www.hiv.lanl.gov

4 http://www.hiv.lanl.gov/content/sequence/HIGHLIGHT/highlighter_top.html

5 http://sray.med.som.jhmi.edu/SCRoftware/simplot/

6 http://www.splitstree.org/

7 http://tree.bio.ed.ac.uk/software/figtree/

8 http://www.hiv.lanl.gov/content/sequence/BASIC_BLAST/basic_blast.html

9 https://coreceptor.geno2pheno.org/

## References

[B1] Addo M. M. Yu X. G. Rathod A. Cohen D. Eldridge R. L. Strick D. (2003). Comprehensive epitope analysis of human immunodeficiency virus type 1 (HIV-1)-specific T-cell responses directed against the entire expressed HIV-1 genome demonstrate broadly directed responses, but no correlation to viral load. *J. Virol.* 77 2081–2092. 12525643 10.1128/JVI.77.3.2081-2092.2003 PMC140965

[B2] Allen T. M. Altfeld M. Geer S. C. Kalife E. T. Moore C. O’sullivan K. M. (2005). Selective escape from CD8+ T-cell responses represents a major driving force of human immunodeficiency virus type 1 (HIV-1) sequence diversity and reveals constraints on HIV-1 evolution. *J. Virol.* 79 13239–13249. 16227247 10.1128/JVI.79.21.13239-13249.2005 PMC1262562

[B3] Apps R. Qi Y. Carlson J. M. Chen H. Gao X. Thomas R. (2013). Influence of HLA-C expression level on HIV control. *Science* 340 87–91. 10.1126/science.1232685 23559252 PMC3784322

[B4] Baryshev P. B. Bogachev V. V. Gashnikova N. M. (2014). HIV-1 genetic diversity in Russia: CRF63_02A1, a new HIV type 1 genetic variant spreading in Siberia. *AIDS Res. Hum. Retroviruses* 30 592–597. 10.1089/AID.2013.0196 24279614 PMC4046202

[B5] Delgado E. Thomson M. M. Villahermosa M. L. Sierra M. Ocampo A. Miralles C. (2002). Identification of a newly characterized HIV-1 BG intersubtype circulating recombinant form in Galicia, Spain, which exhibits a pseudotype-like virion structure. *J. Acquir. Immune Defic. Syndr.* 29 536–543. 11981372 10.1097/00126334-200204150-00016

[B6] Drummond A. J. Rambaut A. (2007). BEAST: bayesian evolutionary analysis by sampling trees. *BMC Evol. Biol.* 7:214. 10.1186/1471-2148-7-214 17996036 PMC2247476

[B7] Fernandez-Garcia A. Delgado E. Cuevas M. T. Vega Y. Montero V. Sanchez M. (2016). Identification of an HIV-1 BG intersubtype recombinant form (CRF73_BG), partially related to CRF14_BG, which is circulating in portugal and spain. *PLoS One* 11:e0148549. 10.1371/journal.pone.0148549 26900693 PMC4765764

[B8] Fernandez-Garcia A. Perez-Alvarez L. Cuevas M. T. Delgado E. Munoz-Nieto M. Cilla G. (2010). Identification of a new HIV type 1 circulating BF intersubtype recombinant form (CRF47_BF) in Spain. *AIDS Res. Hum. Retroviruses* 26 827–832. 10.1089/aid.2009.0311 20618102

[B9] Foster G. M. Ambrose J. C. Hue S. Delpech V. C. Fearnhill E. Abecasis A. B. (2014). Novel HIV-1 recombinants spreading across multiple risk groups in the United Kingdom: the identification and phylogeography of circulating recombinant form (CRF) 50_A1D. *PLoS One* 9:e83337. 10.1371/journal.pone.0083337 24454702 PMC3893077

[B10] Gonzalez-Domenech C. M. Viciana I. Delaye L. Mayorga M. L. Palacios R. De La Torre J. (2018). Emergence as an outbreak of the HIV-1 CRF19_cpx variant in treatment-naive patients in southern Spain. *PLoS One* 13:e0190544. 10.1371/journal.pone.0190544 29309418 PMC5757947

[B11] Han X. An M. Zhang W. Cai W. Chen X. Takebe Y. (2013). Genome sequences of a Novel HIV-1 circulating recombinant form, CRF55_01B, identified in China. *Genome Announc.* 1:e50–12. 10.1128/genomeA.00050-12 PMC3569284 23405298

[B12] Hemelaar J. (2012). The origin and diversity of the HIV-1 pandemic. *Trends Mol. Med.* 18 182–192. 10.1016/j.molmed.2011.12.001 22240486

[B13] Hemelaar J. (2013). Implications of HIV diversity for the HIV-1 pandemic. *J. Infect.* 66 391–400. 10.1016/j.jinf.2012.10.026 23103289

[B14] Hu W. S. Hughes S. H. (2012). HIV-1 reverse transcription. *Cold Spring Harb. Perspect. Med.* 2:a006882. 10.1101/cshperspect.a006882 23028129 PMC3475395

[B15] Huelsenbeck J. P. Ronquist F. (2001). MRBAYES: bayesian inference of phylogenetic trees. *Bioinformatics* 17 754–755. 11524383 10.1093/bioinformatics/17.8.754

[B16] Keele B. F. Giorgi E. E. Salazar-Gonzalez J. F. Decker J. M. Pham K. T. Salazar M. G. (2008). Identification and characterization of transmitted and early founder virus envelopes in primary HIV-1 infection. *Proc. Natl. Acad. Sci. U.S.A.* 105 7552–7557. 10.1073/pnas.0802203105 18490657 PMC2387184

[B17] Kouri V. Aleman Y. Perez L. Perez J. Fonseca C. Correa C. (2014). High frequency of antiviral drug resistance and non-b subtypes in HIV-1 patients failing antiviral therapy in Cuba. *J. Int. AIDS Soc.* 17:19754. 10.7448/IAS.17.4.19754 25397499 PMC4225368

[B18] Kouri V. Khouri R. Aleman Y. Abrahantes Y. Vercauteren J. Pineda-Pena A. C. (2015). CRF19_cpx is an evolutionary fit HIV-1 variant strongly associated with rapid progression to AIDS in Cuba. *EBioMedicine* 2 244–254. 10.1016/j.ebiom.2015.01.015 26137563 PMC4484819

[B19] Kouyos R. D. Von Wyl V. Yerly S. Boni J. Rieder P. Joos B. (2011). Ambiguous nucleotide calls from population-based sequencing of HIV-1 are a marker for viral diversity and the age of infection. *Clin. Infect. Dis.* 52 532–539. 10.1093/cid/ciq164 21220770 PMC3060900

[B20] Kumar S. Stecher G. Tamura K. (2016). MEGA7: molecular evolutionary genetics analysis version 7.0 for Bigger Datasets. *Mol. Biol. Evol.* 33 1870–1874. 10.1093/molbev/msw054 27004904 PMC8210823

[B21] Lai A. Bozzi G. Franzetti M. Binda F. Simonetti F. R. De Luca A. (2016). HIV-1 A1 subtype epidemic in italy originated from africa and eastern europe and shows a high frequency of transmission chains involving intravenous drug users. *PLoS One* 11:e0146097. 10.1371/journal.pone.0146097 26752062 PMC4709132

[B22] Lai A. Bozzi G. Franzetti M. Binda F. Simonetti F. R. Micheli V. (2014a). Phylogenetic analysis provides evidence of interactions between Italian heterosexual and South American homosexual males as the main source of national HIV-1 subtype C epidemics. *J. Med. Virol.* 86 729–736. 10.1002/jmv.23891 24482324

[B23] Lai A. Ciccozzi M. Franzetti M. Simonetti F. R. Bozzi G. Binda F. (2014b). Local and global spatio-temporal dynamics of HIV-1 subtype F1. *J. Med. Virol.* 86 186–192. 10.1002/jmv.23783 24122963

[B24] Lai A. Riva C. Marconi A. Balestrieri M. Razzolini F. Meini G. (2010). Changing patterns in HIV-1 non-B clade prevalence and diversity in Italy over three decades. *HIV Med.* 11 593–602. 10.1111/j.1468-1293.2010.00832.x 20408891

[B25] Lai A. Simonetti F. R. Zehender G. De Luca A. Micheli V. Meraviglia P. (2012). HIV-1 subtype F1 epidemiological networks among Italian heterosexual males are associated with introduction events from South America. *PLoS One* 7:e42223. 10.1371/journal.pone.0042223 22876310 PMC3410915

[B26] Leoz M. Feyertag F. Charpentier C. Delaugerre C. Wirden M. Lemee V. (2013). Characterization of CRF56_cpx, a new circulating B/CRF02/G recombinant form identified in MSM in France. *AIDS* 27 2309–2312. 10.1097/QAD.0b013e3283632e0c 24157908

[B27] Liitsola K. Tashkinova I. Laukkanen T. Korovina G. Smolskaja T. Momot O. (1998). HIV-1 genetic subtype A/B recombinant strain causing an explosive epidemic in injecting drug users in Kaliningrad. *AIDS* 12 1907–1919. 9792392 10.1097/00002030-199814000-00023

[B28] Lukashov V. V. Huismans R. Rakhmanova A. G. Lisitsina Z. N. Akhtyrskaya N. A. Vlasov N. N. (1999). Circulation of subtype A and gagA/envB recombinant HIV type 1 strains among injecting drug users in St. Petersburg, Russia, correlates with geographical origin of infections. *AIDS Res. Hum. Retroviruses* 15 1577–1583. 10580409 10.1089/088922299309874

[B29] Lukashov V. V. Kuiken C. L. Vlahov D. Coutinho R. A. Goudsmit J. (1996). Evidence for HIV type 1 strains of U.S. Intravenous drug users as founders of AIDS epidemic among intravenous drug users in northern Europe. *AIDS Res. Hum. Retroviruses* 12 1179–1183. 8844022 10.1089/aid.1996.12.1179

[B30] Monno L. Brindicci G. Lai A. Punzi G. Altamura M. Simonetti F. R. (2012). An outbreak of HIV-1 BC recombinants in Southern Italy. *J. Clin. Virol.* 55 370–373. 10.1016/j.jcv.2012.08.014 22981618

[B31] Osmanov S. Pattou C. Walker N. Schwardlander B. Esparza J. (2002). Estimated global distribution and regional spread of HIV-1 genetic subtypes in the year 2000. *J. Acquir. Immune Defic. Syndr.* 29 184–190. 11832690 10.1097/00042560-200202010-00013

[B32] Peeters M. D’arc M. Delaporte E. (2014). Origin and diversity of human retroviruses. *AIDS Rev.* 16 23–34. 24584106 PMC4289907

[B33] Posada D. (2008). jModelTest: phylogenetic model averaging. *Mol. Biol. Evol.* 25 1253–1256. 10.1093/molbev/msn083 18397919

[B34] Rawson J. M. O. Nikolaitchik O. A. Keele B. F. Pathak V. K. Hu W. S. (2018). Recombination is required for efficient HIV-1 replication and the maintenance of viral genome integrity. *Nucleic Acids Res.* 46 10535–10545. 10.1093/nar/gky910 30307534 PMC6237782

[B35] Recordon-Pinson P. Alves B. M. Tumiotto C. Bellecave P. Bonnet F. Neau D. (2018). A new HIV-1 circulating recombinant form (CRF98_cpx) between CRF06_cpx and subtype B Identified in southwestern france. *AIDS Res. Hum. Retroviruses* 34 1005–1009. 10.1089/AID.2018.0122 29947242

[B36] Redd A. D. Quinn T. C. Tobian A. A. (2013). Frequency and implications of HIV superinfection. *Lancet Infect. Dis.* 13 622–628. 10.1016/S1473-3099(13)70066-5 23726798 PMC3752600

[B37] Robertson D. L. Sharp P. M. Mccutchan F. E. Hahn B. H. (1995). Recombination in HIV-1. *Nature* 374 124–126. 10.1038/374124b0 7877682

[B38] Rossetti B. Di Giambenedetto S. Torti C. Postorino M. C. Punzi G. Saladini F. (2018). Evolution of transmitted HIV-1 drug resistance and viral subtypes circulation in Italy from 2006 to 2016. *HIV Med.* 19 619–628. 10.1111/hiv.12640 29932313

[B39] Simonetti F. R. Lai A. Monno L. Binda F. Brindicci G. Punzi G. (2014). Identification of a new HIV-1 BC circulating recombinant form (CRF60_BC) in Italian young men having sex with men. *Infect. Genet. Evol.* 23 176–181. 10.1016/j.meegid.2014.02.007 24602697

[B40] Struck D. Roman F. De Landtsheer S. Servais J. Y. Lambert C. Masquelier C. (2015). Near full-length characterization and population dynamics of the human immunodeficiency virus type I circulating recombinant form 42 (CRF42_BF) in luxembourg. *AIDS Res. Hum. Retroviruses* 31 554–558. 10.1089/AID.2014.0364 25654164

[B41] Tebit D. M. Arts E. J. (2011). Tracking a century of global expansion and evolution of HIV to drive understanding and to combat disease. *Lancet Infect. Dis.* 11 45–56. 10.1016/S1473-3099(10)70186-9 21126914

[B42] Thomson M. M. Perez-Alvarez L. Najera R. (2002). Molecular epidemiology of HIV-1 genetic forms and its significance for vaccine development and therapy. *Lancet Infect. Dis.* 2 461–471. 12150845 10.1016/s1473-3099(02)00343-2

[B43] Volz E. M. Le Vu S. Ratmann O. Tostevin A. Dunn D. Orkin C. (2018). Molecular epidemiology of HIV-1 subtype B reveals heterogeneous transmission risk: implications for intervention and control. *J. Infect. Dis.* 217 1522–1529. 10.1093/infdis/jiy044 29506269 PMC5913615

[B44] Wu J. Meng Z. Xu J. Lei Y. Jin L. Zhong P. (2013). New emerging recombinant HIV-1 strains and close transmission linkage of HIV-1 strains in the Chinese MSM population indicate a new epidemic risk. *PLoS One* 8:e54322. 10.1371/journal.pone.0054322 23372706 PMC3553145

[B45] Zhang W. Han X. An M. Zhao B. Hu Q. Chu Z. (2014). Identification and characterization of a novel HIV-1 circulating recombinant form (CRF59_01B) identified among men-who-have-sex-with-men in China. *PLoS One* 9:e99693. 10.1371/journal.pone.0099693 24978029 PMC4076182

