# Supplementary material for: Local Epidemics Gone Viral: Evolution and Diffusion of the Italian HIV-1 Recombinant Form CRF60_BC
Source: Front Microbiol. 2019 Apr 12;10:769. doi: 10.3389/fmicb.2019.00769 (PMC6474184; doi:10.3389/fmicb.2019.00769)
Supplement: Supplementary file 1 [file Image_1.pdf]

Supplementary Material

Local epidemics gone viral: evolution and diffusion of the Italian HIV-1 recombinant form CRF60_BC

Alessia Lai[1]*, Francesco Roberto Simonetti[2], Gaetano Brindicci[3], Annalisa Bergna[1], Simona Di Gianbenedetto[4], Gaetana Sterrantino[5], Cristina Mussini[6], Stefano Menzo[7], Patrizia Bagnarelli[7] Maurizio Zazzi[8], Gioacchino Angarano[3], Massimo Galli[1], Laura Monno[3], Claudia Balotta[1]

*** Correspondence:** Dr Alessia Lai, alessia.lai@unimi.it

Supplementary Figures

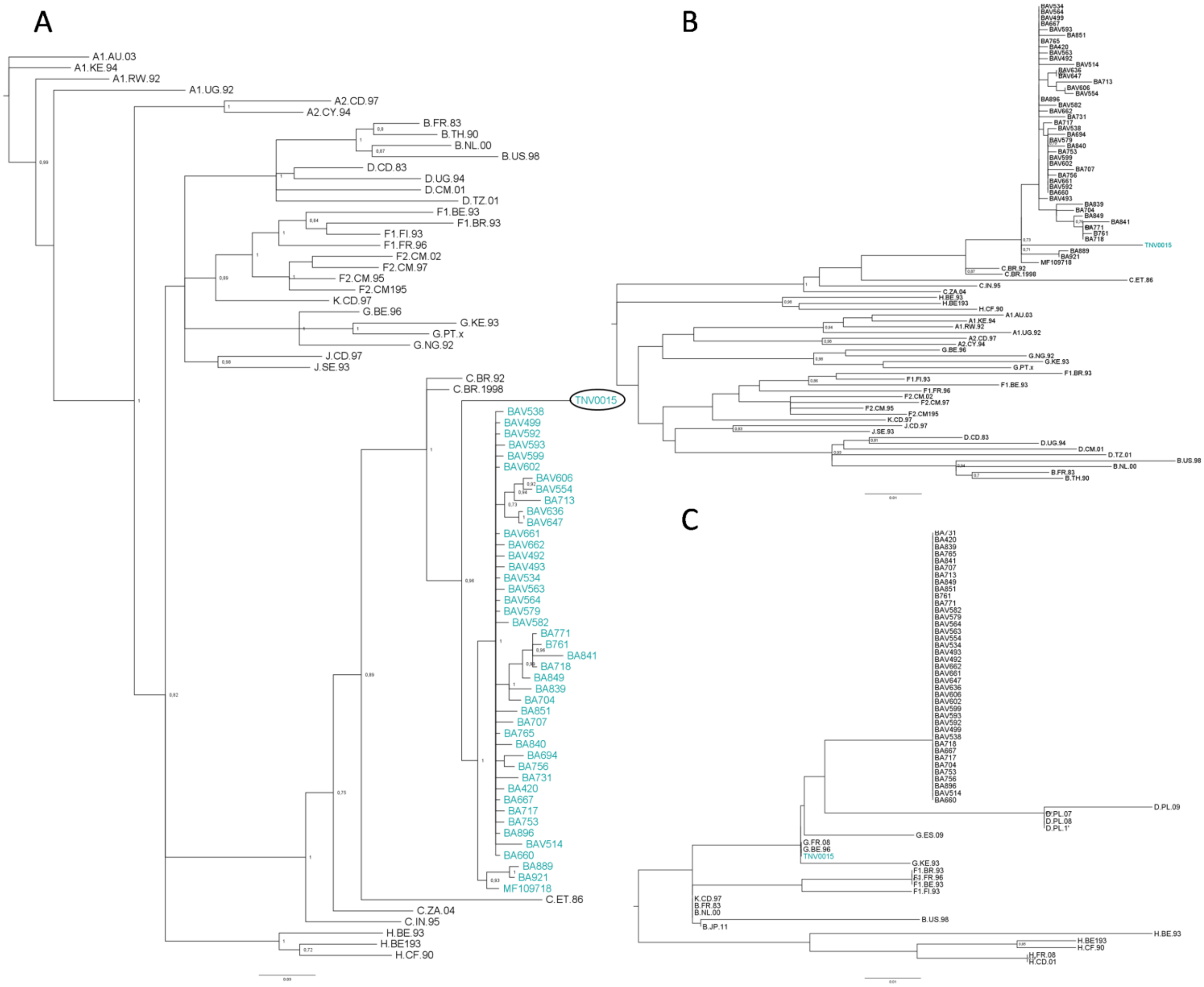

Supplementary Figure 1. Phylogenetic trees of Integrase region showing 2nd generation recombinant (TNV-0015). **(A)** Bayesian tree of CRF60_BC and the unique recombinant obtained using MrBayes. Posterior probabilities >0.7 are shown at nodes. In green the analyzed sequences, the recombinant strain is highlighted with a circle around the name. **(B)(C)** Maximum likelihood trees for the portions identified within breakpoints.

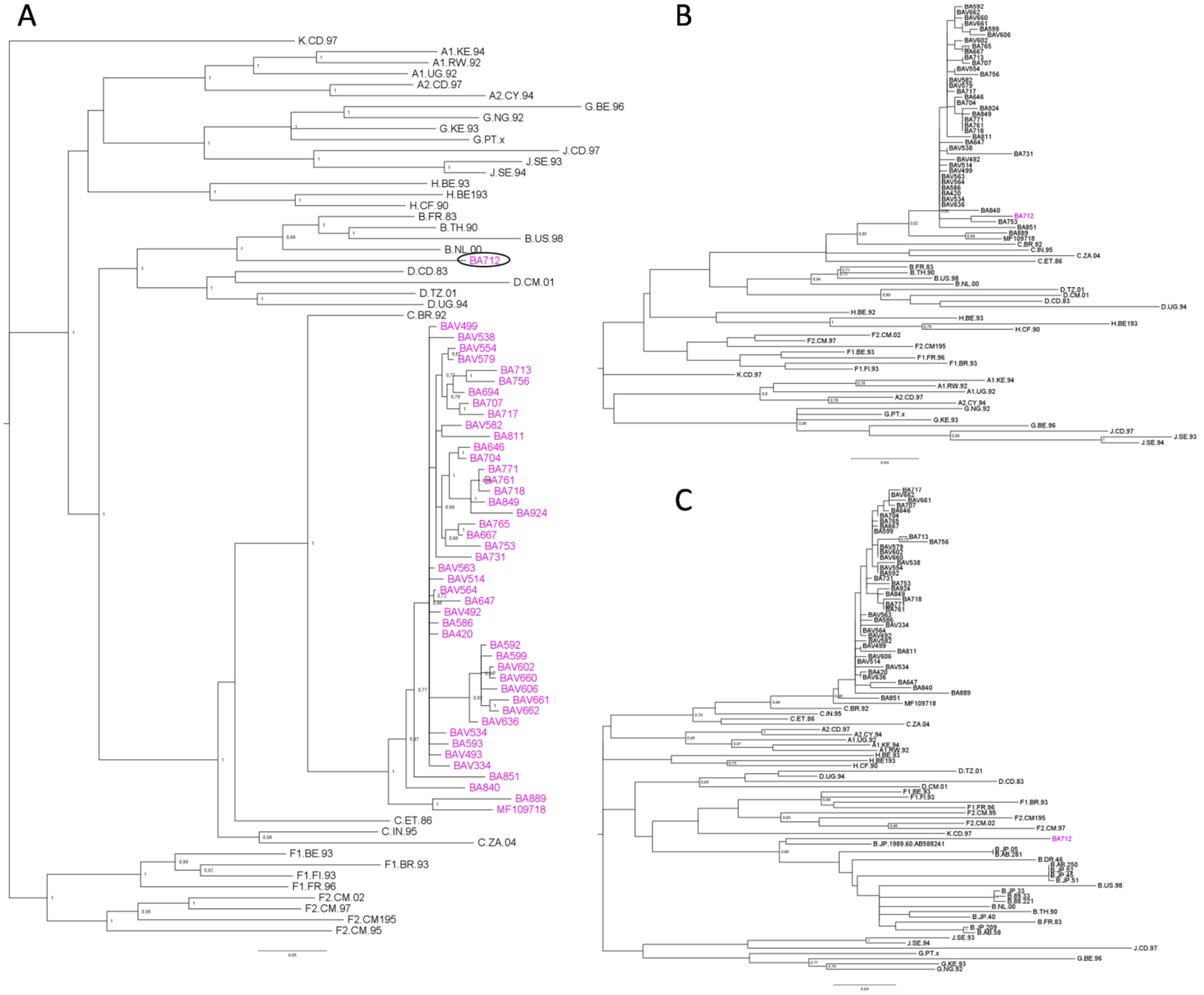

Supplementary Figure 2. Phylogenetic trees of gp120 region showing 2nd generation recombinant (BA712). **(A)** Bayesian tree of CRF60_BC and the unique recombinant obtained using MrBayes. Posterior probabilities >0.7 are shown at nodes. In purple the analyzed sequences, the recombinant strain is highlighted with a circle around the name **(B)(C)** Maximum likelihood trees for the portions identified within breakpoints.

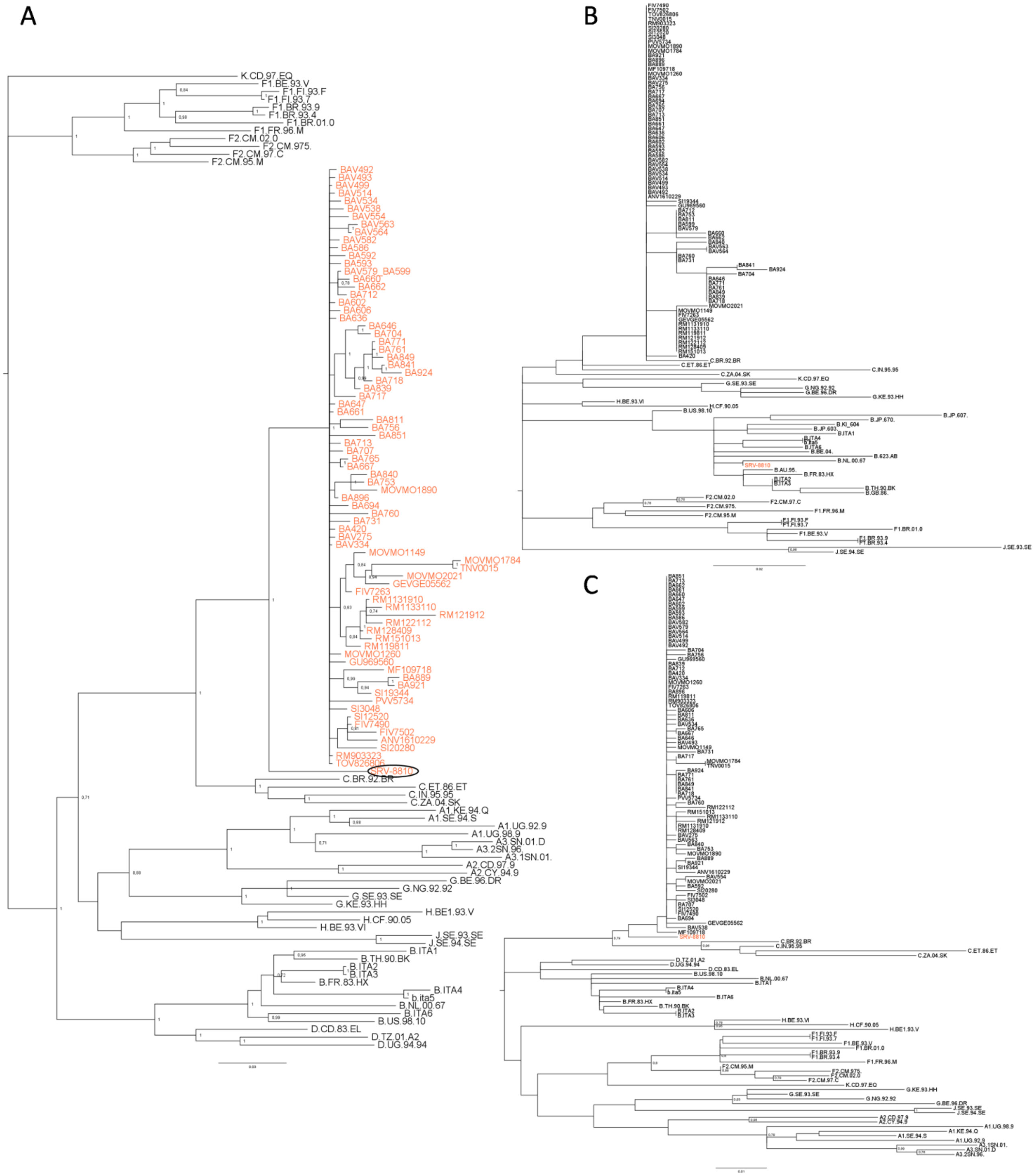

Supplementary Figure 3. Phylogenetic trees of PRO-RT region showing 2nd generation recombinant (SRV8810). **(A)** Bayesian tree of CRF60_BC and the unique recombinant obtained using MrBayes. Posterior probabilities >0.7 are shown at nodes. In orange the analyzed sequences, the recombinant strain is highlighted with a circle around the name. **(B)(C)** Maximum likelihood trees for the portions identified within breakpoints.

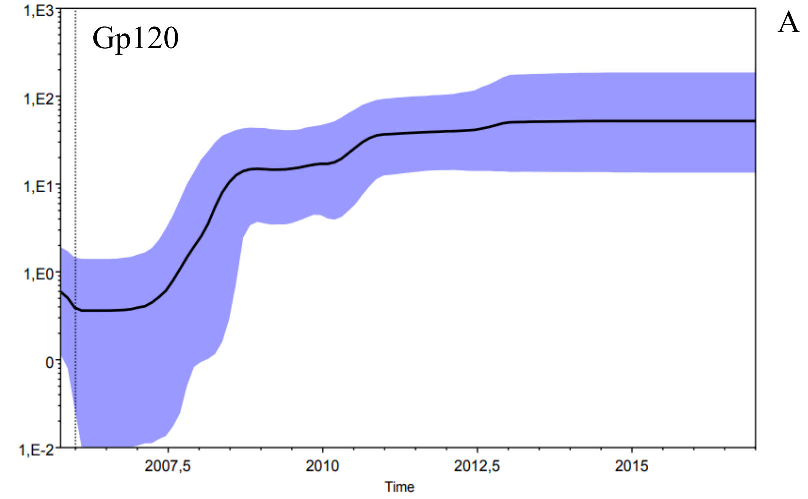

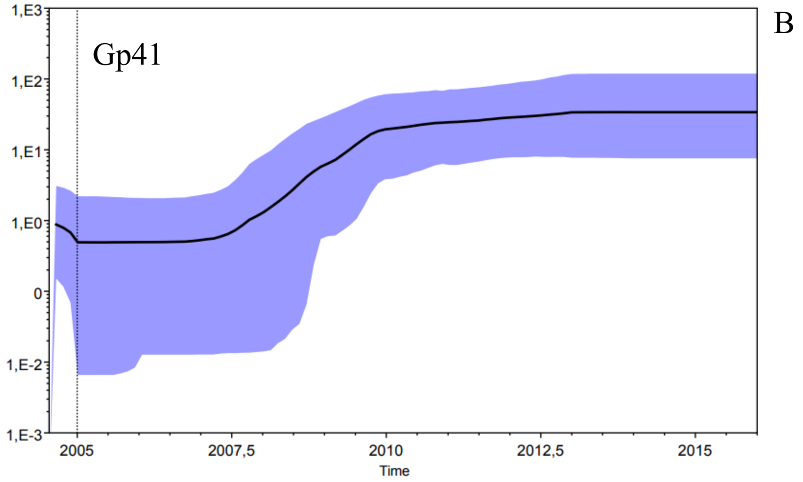

Supplementary Figure 4. Bayesian skyline plot of gp120 and gp41 regions. The curves represent the estimates of the effective number of CRF60_BC infections over time (*x*-axis; calendar year), together with the median estimate (solid line) and 95% HPD confidence interval (blue area). **(A)** Bayesian skyline plot of gp120 region; **(B)** Bayesian skyline plot of gp41 region.
